# Neuroinflammation in Cerebral Ischemia and Ischemia/Reperfusion Injuries: From Pathophysiology to Therapeutic Strategies

**DOI:** 10.3390/ijms23010014

**Published:** 2021-12-21

**Authors:** Anamaria Jurcau, Aurel Simion

**Affiliations:** 1 Department of Psycho-Neurosciences and Rehabilitation, Faculty of Medicine and Pharmacy, University of Oradea, 410087 Oradea, Romania; aurel.simion1962@gmail.com; 2 Neurology Ward, Clinical Municipal Hospital “dr. G. Curteanu” Oradea, 410154 Oradea, Romania; 3 Neurorehabilitation Ward, Clinical Municipal Hospital “dr. G. Curteanu” Oradea, 410154 Oradea, Romania

**Keywords:** ischemic stroke, neuroinflammation, microglia, astrocytes, chemokines, cytokines, stem cells

## Abstract

Its increasing incidence has led stroke to be the second leading cause of death worldwide. Despite significant advances in recanalization strategies, patients are still at risk for ischemia/reperfusion injuries in this pathophysiology, in which neuroinflammation is significantly involved. Research has shown that in the acute phase, neuroinflammatory cascades lead to apoptosis, disruption of the blood–brain barrier, cerebral edema, and hemorrhagic transformation, while in later stages, these pathways support tissue repair and functional recovery. The present review discusses the various cell types and the mechanisms through which neuroinflammation contributes to parenchymal injury and tissue repair, as well as therapeutic attempts made in vitro, in animal experiments, and in clinical trials which target neuroinflammation, highlighting future therapeutic perspectives.

## 1. Introduction

Stroke is the second leading cause of death and a major cause of disability worldwide [1], with an increasing incidence [2] due to demographic changes and the increasing prevalence of diabetes mellitus [3] and obesity [4]. The treatment of ischemic stroke relies increasingly on recanalization strategies, with continuously expanding therapeutic time windows [5,6,7,8]. Unfortunately, reestablishing blood flow in a tissue previously subject to ischemia boosts oxidative stress [9] and leads to the increased release of pro-inflammatory cytokines [10], which trigger a series of pathological cascades which will directly or indirectly cause apoptosis, disruption of the blood–brain barrier (BBB), cerebral edema, and hemorrhagic transformation. Research has shown that neuroinflammatory mechanisms, intimately linked to oxidative stress, significantly contribute to neuronal injury in the acute phase of cerebral ischemia [11], ultimately increasing the magnitude of cerebral damage and neurological deficit [12,13] through ischemia/reperfusion (I/R) injuries. However, in later stages of cerebral ischemia, neuroinflammatory pathways have beneficial effects on tissue repair and functional recovery [14]. As such, unraveling these complex mechanisms and modulating them therapeutically could significantly improve our treatment strategies in ischemic stroke and the quality of life of stroke survivors.

The present paper provides an overview of the involvement of various cell types and pathways in neuroinflammation following acute ischemic stroke and a narrative review of preclinical and clinical studies performed so far (or that are underway) acting on neuroinflammatory mechanisms in an attempt to improve stroke outcome, highlighting promising future perspectives.

## 2. Inflammation in Ischemia/Reperfusion Injuries

The brain is more difficult to access by immune cells from the periphery due to the presence of the BBB [15], which has a layer of endothelial cells interconnected by tight junctions placed on a basal membrane, which embeds a high number of pericytes [16], and ensheathed by astroglial endfeet on the abluminal aspect [17]. However, inflammation, initiated by stagnant blood flow and continued through the activation of intravascular leucocytes and the release of pro-inflammatory mediators from the endothelium and parenchymal cells, is able to substantially potentiate tissue injury [18]. 

It is, nonetheless, important to understand that the neuroinflammatory pathways discussed in the following sections are not merely damaging pathways but also include mechanisms which significantly contribute to reparative processes and enhance recovery after the ischemic insult [14,19]. In fact, research has shown that after acute ischemic stroke, the inflammatory process evolves in three stages [20]: (1) an acute phase in the first hours after stroke onset, during which microglia/macrophages clear the necrotized cells and a first entry of leukocytes, mainly neutrophils, has been described; (2) a subacute phase during the first days after the ischemic insult, associated with a resolution of the inflammatory process; and (3) a late stage, in which the inflammatory cells contribute to astrocytic and microglial reparatory processes. Table 1 summarizes the pathophysiology of cerebral ischemia and reperfusion injuries.

### 2.1. Intravascular Initiation of the Inflammatory Cascade

The inflammatory cascade is activated immediately after vessel occlusion by the modified shear stress on the endothelium and altered blood rheology of stagnant blood flow [21]. P-selectin, stored in Weibel–Palade bodies in endothelial cells and in α-granules in platelets, appears within minutes on the cell surface and, by interacting with P-selectin glycoprotein ligand-1 (PSGL-1) expressed on leucocytes, slows down circulating leucocytes and attracts them to the endothelial surface [18]. Platelet P-selectin also contributes to vascular clogging by binding to leucocytes and facilitating the formation of clusters of leucocytes [21], followed by the activation of coagulation. Inhibition of P-selectin-dependent interaction blocked fibrin deposition in a primate model of thrombosis [22]. 

Activation of the coagulation cascade leads to thrombin generation, which attracts additional monocytes and neutrophils, and induces the expression of adhesion molecules on endothelial cells through the activation of nuclear factor κB (NF-κB) [23]. Thus, intravascular inflammation is the initial step in BBB breakdown and leucocyte infiltration of the ischemic cerebral tissue.

The acute oxygen and glucose deprivation also increases the production of reactive oxygen species (ROS) by cytosolic enzymes and mainly by mitochondria [9], with increased oxidative stress which self-propagates and induces the expression of genes encoding subunits of the α-amino-3-hydroxy-5-methyl-4-isoxazole-propionic acid (AMPA) and kainite glutamate receptors. The consequence of this upregulated gene expression is excitotoxicity, with increases in intracellular calcium concentrations in neurons and to a lesser extent in astrocytes, leading to mitochondrial dysfunction and the initiation of apoptosis [24]. ROS also increase the expression of genes encoding pro-inflammatory factors, such as interleukin (IL) 1β, IL-10, and tumor necrosis factor-α [25], and downregulates the expression of protective genes encoding for subunits of phosphoinositide 3-kinase (PI3K), protein kinase B (Akt), protein kinase C, and calcium/calmodulin-dependent protein kinase II (CAMKII) [25]. Reperfusion, through increasing the oxygen supply, potentiates oxidative stress and its deleterious effects [26]. As such, antioxidants given early after stroke onset could not only increase cell survival but also dampen the inflammatory response elicited by acute ischemia [27].

### 2.2. Parenchymal Inflammation in Cerebral I/R Injuries

#### 2.2.1. Microglia in Neuroinflammation after Acute Ischemic Stroke

The resident immune cells of the brain, representing 5-20% of the glial population, are microglia [28]. They are derived from myeloid progenitors from the yolk sac which seed the cerebral parenchyma during embryonic development [29]. Subsequently, microglia behave similarly to peripheral macrophages despite the latter originating from hematopoietic stem cells [30].

In the resting state, microglial cells have a small cell soma and numerous processes in constant motion which monitor the microenvironment of the CNS [31,32]. The expression of a series of receptors by microglia, such as CD200 receptors, colony-stimulating factor 1 receptors, receptors for chemokines (CX3CL1), neurotrophins, and neurotransmitters [33,34], enable neurons and astrocytes to maintain microglia in a relatively quiescent state [35,36]. 

By expressing Toll-like receptors (TLRs), nucleotide-binding oligomerization domain (NOD)-like receptors (NLRs), and retinoic acid-inducible gene-1 (RIG-1)-like receptors (RLRs), microglia recognize pathogen-associated molecular patterns (PAMPs) and danger-associated molecular patterns (DAMPs), followed by induction of the nuclear factor-kappa B (NF-κB) and Toll/interleukin-1 receptor (TIR)-domain-containing adapter-inducing interferon-β(TRIF)-induced interferon regulatory factor-3 (IRF3) pathways [37,38].

Upon activation by endogenous stimuli generated following injury or infection, microglia retract their processes and take on an amoeboid shape [39,40]. After cerebral ischemia, damaged neurons from the ischemic core release neuromediators, DAMPs, high-mobility group box-1 (HMGB1) protein, and reactive oxygen species (ROS), which activate microglia [41] and the NF-κB pathway. A similar, although more chronic, microglial activation occurs in neurodegenerative diseases as well [42]. Glutamate released by oxygen- and glucose-deprived neurons stimulates the microglial metabotropic glutamate receptor II (mGluRII), leading to the release of TNF-α and activation through the NF-κB pathway [43]. The inhibitory IκB protein, bound to NF-κB in the cytoplasm, is phosphorylated and degraded by IκB kinases, allowing the nuclear translocation of NF-κB, where it promotes the transcription of pro-inflammatory cytokine genes [44,45]. Damaged neurons also release their fractalkine (CX3CL1) ligand, recognized by the microglial CX3CL1 receptor [46].

Once activated, microglia adopt different phenotypes: microglia with enlarged cell bodies with short ramifications and amoeboid cell structures with rare ramifications have been described in the peri-infarct regions in the post-acute phase, while round-shaped microglia, characterizing highly activated cells, have been found near the ischemic core [47]. Aside from the morphological changes, microglia also exhibit different gene expression patterns with distinct functions. Classically, the M1 phenotype is pro-inflammatory while the M2 phenotype has anti-inflammatory actions [48], with M2a phenotypes being involved in reparative and regenerative processes, M2b phenotypes displaying immunoregulatory properties, and M2c microglia clearing cellular debris [49]. M2a activation is induced by IL-4 and IL-13 [50,51]. M2b polarization has been observed with the triggering of TLRs and Fcγ receptors (FcγR—receptors for the Fc part of IgG), while M2c polarization has been described in response to anti-inflammatory factors such as IL-10, transforming growth factor-β (TGF-β), and glucocorticoids [40,52]. Chemokines, such as CCL2 and CXCL4, can also induce M2 polarization [53,54]. The various microglial phenotypes can be identified through their surface markers as well. M1 phenotypes express CD11b, CD45, CD68, CD16, CD32, FcγR, and iNOS (inducible nitric oxide synthase) [55], while M2 phenotypes express CD206 (mannose receptor), arginase-1, and Ym1 (chitinase-like-3, Chi3l3), which prevent the degradation of extracellular matrix components [56].

Once activated and polarized towards an M1 phenotype, microglia release tumor necrosis factor-alpha (TNF-α), inflammatory interleukins such as IL-1β, IL-6, other inflammatory cytokines, and reactive oxidative species (ROS) into the circulation [57,58]. The cytokines and chemokines produced by microglia can recruit leukocytes to the injured parenchyma [59], which will release proteases and oxygen radicals, thereby potentiating tissue destruction [43]. 

M2-polarized microglia, by producing anti-inflammatory factors (IL-4, IL-10, IL-13, or TGF-β), contribute to the resolution of inflammation [60] and have important roles in tissue remodeling and repair as well as angiogenesis [61,62] by producing insulin-like growth factor-1 (IGF1), which suppresses apoptosis and increases proliferation and differentiation of neural precursor cells (NPCs) [56], brain-derived neurotrophic factor (BDNF), and neuronal growth factor (NGF) [63]. 

Being highly plastic cells, microglia can shift between different phenotypes [64,65], as demonstrated in an experimental model of autoimmune encephalomyelitis [66], illustrating the dual role of inflammation after a tissue injury [67]. 

Temporal analyses of microglial phenotypes in animal stroke models showed an increase in the M1 phenotype in the first 14 days post-stroke, while M2 phenotypes were detected 12 h after the ischemic insult and increased during the first 3 days, followed by a decline in their number [55]. 

After transient middle cerebral artery occlusion in mice, microglial and macrophage infiltration peaks at 48-72 h after the ischemic insult [68]. Transient middle cerebral artery occlusion as short as 15 min in spontaneously hypertensive stroke-prone rats led to microglial activation [69], after which these cells migrated toward the ischemic lesion and remained close to the neurons in a process called “capping”, which contributed to rapid removal of damaged neurons [28,70]. The production of ROS (mainly via NADPH oxidase (NOX)), matrix metalloproteinases, and cytokines, as well as activation of CD14 receptors by iNOS followed by the expression of TLR4 in activated microglia, increased its neurotoxic effects in the infarcted core as well as in the penumbra [71,72,73]. TLR4 depletion in mice reduced the magnitude of ischemic damage [74], as did inhibition of microglial activation (with 2% isoflurane) in rats subjected to transient focal cerebral ischemia [75]. However, the ablation of proliferating activated microglia impaired the production of IGF1 within the injured tissue [76], while transplantation of cultured microglia into the ischemic brain diminished the extent of ischemic damage and enhanced functional recovery [77]. 

In humans, histological studies and, more recently, positron emission tomography studies have shown abundant activated microglia in the ischemic core within 24-48 h after stroke onset [78], which persisted for several weeks, mainly at the periphery of the ischemic core [79]. Activated microglia were also detected in the thalamus ipsilateral to the infarcted hemisphere 7 days after stroke onset [80], a finding ascribed to the secondary degeneration of fibers. The persistence of activated microglia in the subacute phase of stroke in the penumbra makes the pharmacological manipulation of microglia an attractive possibility [81]. In the chronic phase, M2-polarized microglia, by producing a variety of neurotrophic factors, promote neuroplasticity and neurogenesis [82]. 

#### 2.2.2. Astrocytes in Post-Stroke Neuroinflammation

Astrocytes are essential housekeeping cells supporting neuronal function by regulating the ion–water balance; removing excess neurotransmitters and waste products; secreting trophic factors such as fibroblast growth factor-2, brain-derived neurotrophic factor, and nerve growth factor; and, through their end feet, participating in the normal structure and function of the BBB [83]. 

Following ischemia, the impaired expression of the excitatory amino acid transporter 2 (EAAT2) interferes with the clearance of glutamate by astrocytes [84], while cytokines derived from neurons and glial cells lead to astrocytic hyperplasia and activation [85]. Activated astrocytes release vimentin, Il-1β, monocyte chemotactic protein-1, and glial fibrillary acidic protein (GFAP) and contribute to the formation of glial scars [86] as well as MMP-2, which weakens the BBB [87]. 

In animal experiments, astrocytic activation started 4 h after the insult, peaked on day 4, and persisted for 28 days [88]. An important cytokine whose expression is increased in astrocytes after ischemia is IL-15 [28]. Transgenic mice expressing IL-15 with GFAP promoter exhibited worse neurological deficits following cerebral ischemia [28], while IL-15 knockdown in mice diminished the infarct size [89]. 

#### 2.2.3. Leukocytes in Neuroinflammation after Acute Ischemic Stroke

**Figure 1:**
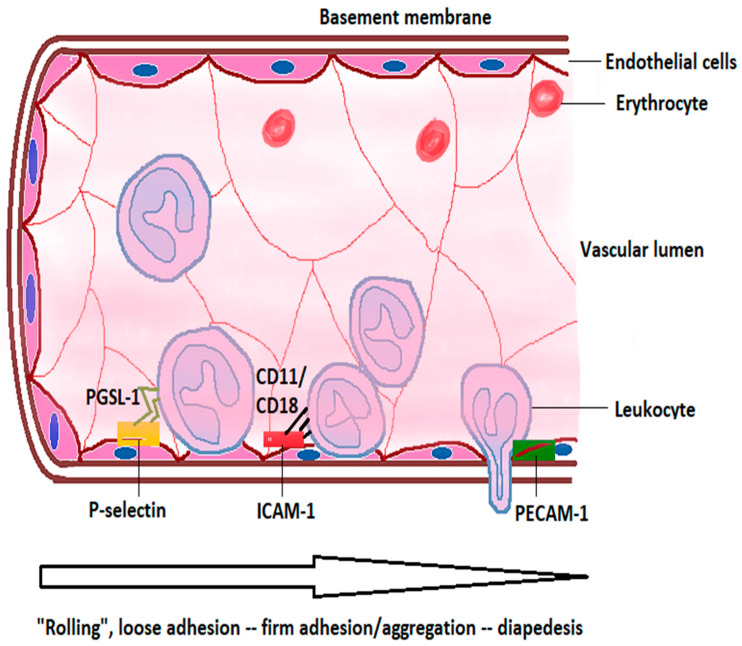
Leukocyte diapedesis. Leukocytes interact with endothelial cells expressing P-selectins through P-selectin glycoprotein 1 (PSGL-1), leading to their “rolling” on the endothelial surface. Interaction of leukocyte integrins CD11a/CD18 and CD11b/CD18 with intercellular adhesion molecule 1 (ICAM-1) leads to firm adherence and aggregation of leukocytes. Diapedesis of leukocytes is facilitated by the expression of platelet endothelial cell adhesion molecule 1 (PECAM-1) by endothelial cells. Adapted from Collard and Gelman [96].

Leukocytes, mainly neutrophils [90], are among the first blood-derived immune cells entering the brain after cerebral ischemia, peaking at 48-72 h and rapidly declining afterwards [18]. Minutes to hours following the ischemic insult, ROS, cytokines, and chemokines released by the damaged tissue induce the expression of adhesion molecules on leukocytes and cerebral endothelial cells [28,91]. Cytokines such as TNF-α or IL-1β lead to the translocation of P-selectin to the nucleus and its expression on the endothelial cells, which, by interaction with its receptor, PSGL-1, on leukocytes, slows the latter down, leading to leukocyte “rolling” on the endothelium. Further, the expression of intercellular adhesion molecule (ICAM)-1 and vascular cell adhesion molecule (VCAM)-1 on the endothelial cell surface and their interaction with the leukocyte β2 integrins CD11a/CD18 and CD11b/CD18 lead to their firm adhesion and aggregation on the endothelial surface [15,91,92]. The leukocytes change their shape, become flattened, and redistribute adhesion, signaling, and receptor proteins toward an edge from which processes extend [26]. The expression of platelet endothelial cell adhesion molecule-1 (PECAM-1) along the endothelial cell junction, as well as the expression of the junctional proteins JAM-A and JAM-B by pericytes, facilitates neutrophil diapedesis across the BBB [93,94], as illustrated in Figure 1. Once in the tissue, leukocytes produce a series of factors which exacerbate tissue injury, such as ROS, proteases, IL-1, IL-6, IL-12, and TNFα [26,95]. 

The various subtypes of leukocytes which infiltrate the brain parenchyma after an ischemic stroke contribute to cerebral injury in many ways. Adherence of leukocytes to the endothelium can block erythrocytes in the microvasculature (“clogging”), leading to the “no-reflow” phenomenon [97]. In addition, activated leukocytes produce ROS, proteases, and matrix metalloproteinases (especially MMP-9), which damage the blood vessel wall and surrounding brain tissue [98]. Phospholipases in activated leukocytes produce biologically active substances such as prostaglandins, leukotrienes, and eicosanoids, which lead to vasoconstriction and platelet aggregation. In addition, by releasing pro-inflammatory factors, infiltrated leukocytes further promote neuronal injury [28,99]. 

Among the leukocytes infiltrating the brain are blood-derived monocytes/macrophages, which infiltrate the cerebral tissue in the hyperacute stage of stroke (<24 h) [100]. They have similar functions to resident microglia and are very difficult to differentiate from the latter [43], with researchers using chimera mice with enhanced colored fluorescent protein bone marrow for this aim [101]. Blood-derived macrophages peak by day 7, followed by a decline in their number.

Neutrophils start infiltrating the ischemic tissue after 30 min and peak between days 1 and 3, after which their number declines [102]. In the first 3 days following ischemia, neutrophils are the predominant inflammatory cells present in ischemic tissue [68], and their presence has been positively correlated with infarct size and neurological functional impairment [102]. 

Lymphocytes have less important contributions in cerebral ischemic injury; the mechanisms are mainly related to the innate T cell functions [103]. IL-17 secreting γδT cells, CD4^+^ T cells, and CD8^+^ T cells may infiltrate the parenchyma within hours after stroke onset, peak by day 3, and aggravate ischemic injury [68,103,104], as do natural killer T cells [105], while regulatory lymphocytes (Tregs) expressing the forkhead box P3 (Foxp3) transcription factor [106,107] have traditionally been regarded as exhibiting neuroprotective activity [108,109]. The choroid plexuses appear to be the preferential route for infiltration by lymphocytes since in an experimental setting, infarction of the choroid plexus resulted in diminished lymphocyte infiltration [110]. 

In the early post-stroke phase, Tregs act on peripheral leucocytes to inhibit the production of matrix metalloproteinase-9 (MMP-9) by neutrophils [111,112], thereby protecting the blood–brain barrier, and prevent the activation of effector T cells by secreting anti-inflammatory IL-10 and TGF-β [113]. However, by having a higher adhesive propensity, natural Tregs interact in the hyperacute phase of ischemic stroke with activated endothelial cells and platelets, leading to microvascular dysfunction and thrombus formation, resulting in impaired reperfusion [114]. Selective depletion of Tregs in mice significantly reduced infarct size and improved functional recovery [114] in a transient middle cerebral artery occlusion model. In later stages, 5 to 7 days after stroke onset, Tregs infiltrate the brain parenchyma, prevent microglia/macrophage polarization toward the M1 phenotype, driving microglial polarization toward the M2 phenotype, and inhibit astrocytic activation through the amphiregulin (AREG)/epidermal growth factor receptor (EGFR) pathway [115,116]. In addition, through producing IL-10, which interacts with the IL-10 receptor expressed on neural stem cells, Tregs promote the proliferation of these cells in the subventricular zone as well as the migration of neural progenitor cells to the injured area and their differentiation into mature neurons [117]. 

#### 2.2.4. Platelets in Cerebral Ischemia/Reperfusion Injury

During ischemia and energy failure, there is a build-up of hypoxanthine which, upon reperfusion, will be converted by hypoxanthine oxidase to xanthine and uric acid, with significant amounts of superoxide generated in the process [118]. In addition, dysfunctional mitochondria and leukocyte NADPH oxidases also produce ROS during reperfusion [118], which can activate platelets and enhance their activity by quenching NO and decreasing cyclic guanosine monophosphate and protein kinase G activity inside the platelets [119,120,121]. This leads to altered calcium signaling and platelet activation, adhesion, and aggregation [119,120], as well as vasoconstriction (also caused by limited availability of NO), both of which are involved in the no-reflow phenomenon following reperfusion [122]. 

In response to hypoxia, and additionally stimulated by uric acid [123], endothelial cells release von Willebrand factor (VWF) stored in Weibel–Palade bodies into the media. Activated platelets are additional sources of VWF. VWF is secreted as large, highly thrombotic polymers cleaved into smaller fragments by disintegrin-like and metalloprotease with thrombospondin type 1 motif number 13 (ADAMTS 13) [124]. Genetic deletion of ADAMTS 13 resulted in increased infarctions after experimentally induced transient middle cerebral artery occlusion [125]. VWF contributes to platelet activation by binding glycoprotein Ib (GPIb) [119] and is also involved in leukocyte recruitment [126]. Recruited leukocytes phagocytose degraded cells, release supplemental amounts of ROS [127], and form complexes with platelets (platelet–neutrophil aggregates) which are implicated in capillary no-reflow as well, along with pericyte contraction [128,129]. 

A series of molecules released by damaged cells (DAMPs, HMGB1) interacts with platelet TLR 4 and promotes platelet activation and thrombus formation [130]. In addition, platelets can bind to activated endothelial cells via cell adhesion molecules (ICAM, VCAM), CD40–CD40L interactions, and selectins expressed by endothelium [131]. Electron microscopy studies have shown that following reperfusion endothelial denudation is worse compared to ischemia, creating the premises for platelets to interact with exposed collagen [132]. 

After activation, the content of platelet granules is released and serves to further increase platelet activation (adenosine diphosphate) or act as pro-inflammatory cytokines (such as platelet factor 4 and β-thromboglobulin) to activate endothelial cells and recruit additional leukocytes and form platelet–neutrophil aggregates [133]. Phosphatidylserine is exposed and mediates the conversion of prothrombin to thrombin by recruiting the coagulation factors Xa and Va [134].

However, platelet granules also contain factors which prevent the aggravation of ischemia/reperfusion injuries, especially hemorrhagic transformation [135]. Platelet inhibition with monoclonal antibodies such as abciximab, or with the synthetic molecule tirofiban, resulted in bleeding complications [136]. Moreover, platelets also release anti-inflammatory cytokines, such as IL-10 [137], as well as brain-derived neurotrophic factor (BDNF), stored in granules and platelet cytosol and released after the activation of thrombin receptors PAR1 and PAR4 [138]. After release, BDNF promotes neurogenesis, oligodendrogenesis, and remyelination of axons in the white matter [90,139]. 

### 2.3. Inflammatory Mediators

#### 2.3.1. Cytokines

Cytokines are small polypeptides (8-26 kDa), normally expressed at very low levels, which regulate immune responses [140]. Various cytokines can either mediate the inflammatory response or act as anti-inflammatory molecules, which terminate the inflammatory response.

##### Pro-Inflammatory Response

Necrotic neurons release DAMPs, or “hazard signals”, which induce TLR4 on microglia and stimulate microglial cells to produce inflammatory cytokines via the NF-κB pathway [141]. 

M1-polarized microglia will produce pro-inflammatory cytokines such as IL-1β, IL-6, IL-18, and TNF-α [141]. The perivascular macrophages, lying between the brain surface and the vascular endothelial membrane, produce IL-1β, IL-12, IL-23, TNF-α, chemokines, and ROS [142]. T helper cells, once in the brain parenchyma, release ROS, IFN-γ, TNF-α, IL-1β, IL-17, and IL-21, damaging the neurons and neurovascular unit [143], while natural killer T cells are neurotoxic by releasing IL-2 and TNF-α [142]. 

The best-studied is the NLRP3 inflammasome, activated in two steps. First, DAMPs activate TLRs on immune cells, which induce the expression of inactive protein NLRP3 and pro-IL-1β via the NF-κB pathway [144,145]. Resting NLRP3 is situated at the endoplasmic reticulum. In step two, an effector domain (pyrin domain, or PYD) is oligomerized in the NLRP3 central domain, serving as a girder for ADC adaptor protein (apoptosis-associated speck-like protein containing a carboxy-terminal CARD), which combines with procaspase-1 and forms the NLRP3 inflammasome [146]. This, in turn, by using the CARD (caspase activation and recruitment domain), activates procaspase-1 to caspase-1 and splits pro-IL-1β into its active form [147], leading to pyroptosis, a specific type of cell death [141]. 

IL-1 occurs in 3 forms: IL-1α, IL-1β, and IL-1ra [85]. IL-1α is intracellular, while IL-1β is secreted extracellularly. IL-1ra is the antagonist of IL-1β, the expression of which is induced by very high levels of IL-1β [85,148]. Most researchers reported increased levels of IL-1β within 24 h after stroke onset [149], while others did not [150]. IL-1β exacerbates cerebral injury after an ischemic insult and significantly contributes to the disruption of the BBB. IL-1β administered to rats increased the magnitude of brain injury [151], while IL-1β-deficient mice had smaller-volume infarcts compared with wild-type mice [152]. In clinical setting, patients treated with the recombinant human IL-1 receptor antagonist (IL-1Ra, or anakinra) had reduced circulating pro-inflammatory markers (TNFα, IL-6, and IL-10) and better clinical outcome as compared to placebo [153]. The usefulness of anakinra in downregulating the inflammatory response was further proved in the recent COVID-19 pandemic [154,155]. 

Tumor necrosis factor (TNF)-α has two forms: a transmembrane form, involved in regulation of inflammation via cell–cell interactions (tmTNF-α), and a soluble form (sTNF-α), which increases the phagocytic and cytotoxic activity of macrophages and upregulates the expression of IL-1β and IL-6 [142,156,157,158]. TNF-α is upregulated after cerebral ischemia, and protein levels are increased by 3 h after ischemia, peaking up to 5 days after the insult [159]. The cytokine induces the release of matrix metalloproteinase-9 (MMP-9) from pericytes, leading to increased permeability of the BBB [160]. However, other researchers have shown TNF-α to be neuroprotective and involved in ischemic preconditioning [161]. Thus, it appears that the dual role depends on the source of TNF-α, with microglial-derived TNF-α being neuroprotective in stroke [140]. 

Both IL-1β and TNF are required for the synthesis of IL-6, released by microglia, astrocytes, neurons, and endothelial cells [162]. Within a few hours after stroke onset, there is a rise in the concentration of IL-6 [163], which correlates well with stroke severity and worse prognosis [164], although other researchers have found opposite results [163]. When analyzing the different stroke subtypes, it appeared that embolic strokes had the highest IL-6 levels [165].

IL-8 also promotes inflammatory responses in cerebral ischemia, proven by the reduced infarct size and better outcome after blocking the cytokine and its receptors [166,167].

Interferon gamma (IFN-γ) is another cytokine able to modulate immune responses and produced by various cell types, such as monocytes, macrophages, and B and T lymphocytes, as well as natural killer cells [85]. It can activate multiple downstream signaling cascades, such as the Janus kinase (JAK)-signal transducer and activator of transcription (STAT) pathway, and modulates microglial polarization [85].

Other pro-inflammatory cytokines are IL-16, IL-17, IL-18, monocyte chemotactic protein 1 (MCP-1), and leukotrienes [13]. 

##### Anti-Inflammatory Cytokines

In the subacute phase, M2-polarized microglia start producing anti-inflammatory cytokines, such as IL-10, IL-4, and transforming growth factor-beta (TGF-β), which will lead to inhibition of the inflammatory response [144].

IL-10 is an anti-inflammatory cytokine produced by various cell types, such as monocytes, CD4, CD25, Foxp3 regulatory T cells, and mastocytes [168]. IL-10 is upregulated in ischemic stroke, peaking 3 days after the onset [169]. It can counterbalance the actions of IL-1β and TNF-α, protecting against injury in ischemic stroke [13,170]. Animal research with intraventricular administration of IL-10 or adenoviral delivery of the IL-10 gene confirmed the neuroprotective effect of this cytokine [171,172]. In human patients, low plasma levels of IL-10 in the first hours after stroke onset were associated with worse neurological deficits at 48 h [173]. 

While IFN-γ is a pro-inflammatory cytokine, interferon-β (IFN-β) has been used as immunomodulatory treatment in multiple sclerosis, where it reduces MMP-9 levels and diminishes BBB disruption [174,175]. In an experimental setting, IFN-β downregulated ICAM-1 expression on cerebral endothelial cells and attenuated BBB disruption and neutrophil infiltration in a rat model of stroke, thereby reducing infarct volume [176,177]. 

Transforming growth factor-beta (TGF-β), although rarely expressed in normal brain, has been found significantly upregulated after stroke, being produced by microglia, macrophages, neurons, and astrocytes [140]. TGF-β3 is especially involved in neuronal survival and tissue recovery [85]. In an experimental setting, antagonizing TGF-β resulted in increased infarct volume [178], while intranasal administration of TGF-β reduced infarct volume and enhanced neurogenesis after ischemic stroke in mice [179]. 

IL-4 is able to polarize microglia toward the anti-inflammatory M2 phenotype [180], indirectly supporting cell survival and tissue repair [181]. 

#### 2.3.2. Chemokines

Chemokines, or chemotactic cytokines, are low-molecular-weight proteins (8-10 kDa) with an *N*-terminal domain, a β-sheet subunit, and a C-terminal α-helix that are involved in cellular activation and leukocyte recruitment. Their main function is to control leukocyte migration into tissues during inflammation [141]. Depending on the number and location of cysteine residues, they can be divided into [182]:

- CC—two cysteine residues, immediately adjacent,
- CXC—two cysteine residues separated by one amino acid,
- CX3C—two cysteine residues separated by three amino acids.

Chemokines are released by neurons, astrocytes, microglial cells, oligodendrocytes, and endothelial cells [183] and act under normal conditions mainly as trophic and protective modules in the nervous system. Their role in ischemic stroke pathogenesis is still controversial. 

Monocyte chemoattractant protein-1 (MCP-1) directly increases the permeability of the BBB by causing tight junction proteins to redistribute in endothelial cells [184] and also recruits monocytes and activated lymphocytes into the brain after an ischemic insult [185]. Mice lacking MCP-1 receptors have reduced infarct size, reduced BBB permeability, and diminished edema compared to wild-type mice following transient focal cerebral ischemia [186]. 

Other chemokines upregulated in the first 3 h after stroke are microglial response factor-1 (MRF-1), fractalkine (CX3CL1), and macrophage inflammatory protein 1 (MIP-1), which all contribute to the infiltration of the injured tissue with inflammatory cells and thus weaken the BBB [22]. However, the role of fractalkine is still under investigation, with research pointing towards a dual role, being both neuroprotective and pro-inflammatory [184,185]. A knock-out of CX3CL1 and CX3CL1 receptors resulted in reduced lesion volume after middle cerebral artery occlusion [187]. On the other hand, exogenous fractalkine administered shortly before middle cerebral artery occlusion to wild-type animals resulted in diminished size of the subsequent lesion, increased lesion size in CX3CL1 knock-out animals, and had no effect on animals lacking CX3CL1 receptors [188]. It appears that the neuroprotective effect of fractalkine depends on its interaction with microglia [184]. CCL5 is another chemokine studied, with research revealing reduced infarct volume in a middle cerebral artery occlusion in CCL5 knock-out mice [189].

Stromal cell-derived factor 1 (SDF-1), also known as CXC motif chemokine 12 (CXCL12), also seems to have a dual role. SDF-1 and its receptor were found upregulated in the penumbral area [190], and rats treated with antagonists of SDF-1 receptors had improved functional outcome after stroke [191]. However, other researchers emphasized the neuroprotective role of SDF-1, showing that it facilitates the homing of bone marrow stromal cells to the tissue injured by ischemia [192], thereby reducing the size of infarction and enhancing neural plasticity [193]. 

#### 2.3.3. Matrix Metalloproteinases

Although not inflammatory mediators, matrix metalloproteinases (MMPs) are tightly related to neuroinflammation and contribute significantly to BBB disruption, cerebral edema, and hemorrhagic transformation. MMPs are a family of proteolytic enzymes which have a catalytic zinc site, a pro-peptide region, a fibronectin binding site, and a transmembrane site [28]. They can be either constitutive, acting close to the site of activation (MMP-2, MMP-14), or inducible (MMP-3, MMP-9), acting at distant sites [194]. Although traditionally regarded as pro-inflammatory enzymes with deleterious effects in stroke pathophysiology, research has shown that they are also involved in neuronal regeneration, angiogenesis, and cell proliferation [195]. 

Upon activation of the constitutive MMPs, such as MMP-2, they fragment the tight junction proteins which “zip” endothelial cells, leading to a transient opening of the BBB 2 to 3 h after reperfusion [196,197]. After this early phase of BBB dysfunction, its integrity is restored until 24-48 h after the ischemic insult, when a second opening of the BBB is described with more dramatic consequences, such as cerebral edema, hemorrhagic transformation, and leukocyte infiltration, due to the expression and activation of inducible MMPs, such as MMP-3 and MMP-9 [198]. MMP inhibitors were able to reduce infarct size and edema in animal models of stroke, but their clinical use is hampered by their pharmacokinetic profile and significant toxicity [198,199]. Inducible MMPs, being free to move in the extracellular space, are able to inflict greater damage on the extracellular matrix and basal lamina [28]. Further, tissue plasminogen itself, if given during the early opening of the BBB, is able to get access into the cerebral parenchyma and upregulate inducible MMPs, which will lead to BBB damage with hemorrhagic complications and edema [196]. 

On the other hand, plasma levels of MMP-3 were found increased in patients with better functional and motor recovery [200], highlighting the dual role of these enzymes in stroke pathogenesis and recovery, while 1-2 weeks following stroke onset, MMP-9 is upregulated in the peri-infarct zone and colocalizes with markers of neurovascular remodeling [201], a process which likely involves the processing of VEGF (vascular endothelial growth factor). MMP-9 is able to transform pro-VEGF and matrix-bound VEGF into active molecules [202]. In fact, delayed inhibition of MMPs, starting with day 7, worsened outcome after stroke [201], suggesting that modulation of MMPs instead of blocking them would be a more rewarding approach.

**Figure 2:**
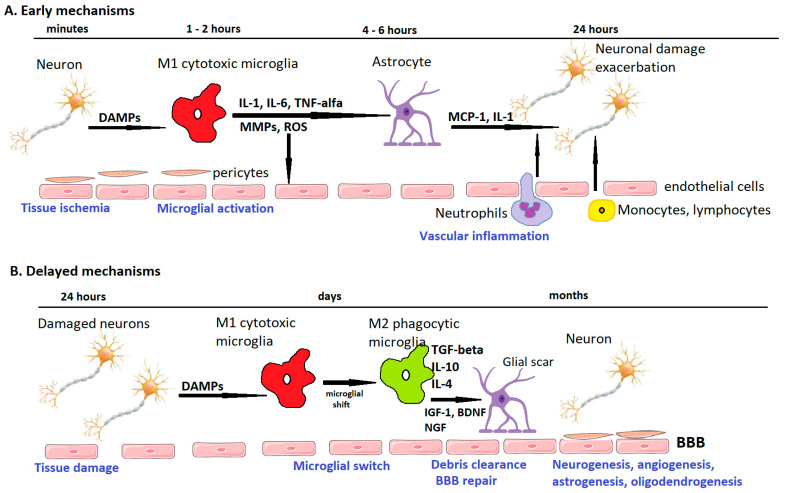
Temporal profile of post-stroke ischemic pathways. Immediately after ischemic stroke, neurons release damage-associated molecular patterns (DAMPs) which lead to microglial and endothelial activation. M1-polarized microglia release pro-inflammatory interleukins (IL-1, IL-6, tumor necrosis factor-alpha), as well as matrix metalloproteinases (MMPs) and reactive oxygen species (ROS), which weaken the blood–brain barrier (BBB). Pericytes and astrocytic endfeet are lifted from the basement membranes. A “leaky” BBB allows leukocytes to infiltrate the cerebral parenchyma, where they produce pro-inflammatory factors (IL-1, monocyte chemotactic protein 1 (MCP-1)) and exacerbate tissue injury. In the delayed subacute phase, microglial switch to an M2 phenotype leads to tissular debris clearance, and, by expressing anti-inflammatory mediators and neurotrophic factors such as insulin-like growth factor 1 (IGF-1), brain-derived neurotrophic factor (BDNF), or nerve growth factor (NGF), promotes glial scar formation as well as BBB repair, neurogenesis, astrogenesis, oligodendrogenesis, and angiogenesis. Adapted from Rajkovic et al. [204].

Table 2 summarizes the beneficial and detrimental effects of the main inflammatory molecules in ischemic stroke, and Figure 2 schematically presents the temporal profile of the neuroinflammatory pathways.

**Table 2:** Beneficial and detrimental effects of the main inflammatory mediators. Adapted from Jayaraj et al. [28].

Inflammatory Mediators	Beneficial Effects	Detrimental Effects	References
TNF-α	Stimulates the expression of antioxidants and anti-apoptotic factors, involved in ischemic preconditioning, increases expression of neurotrophic factors, modulates neuronal plasticity	Increases infarct volume, promotes leukocyte adherence to endothelium, contributes to BBB disruption, edema formation, increases apoptosis of endothelial cells	[15,28,91,92,152,160,161,203]
IL-6	Enhances post-stroke angiogenesis	Contributes to leukocyte recruitment and promotes neuroinflammation, increases stroke severity	[28,164]
IL-8		Augments neuroinflammation	[28,166,167]
IL-10	Diminishes cytokine release, promotes neuronal survival and neurogenesis, promotes M2 polarization of microglia/macrophages		[28,40,52,117]
IL-1β		Disruption of BBB, edema formation, leukocyte recruitment	[28,59,151]
MMPs	Promote vascular remodeling	BBB disruption, vasogenic edema, hemorrhagic transformation	[28,196,201]
Interferon-β	Downregulates ICAM-1 expression, attenuates BBB disruption		[176,177]
MCP-1		Increases BBB permeability, enhances leukocyte infiltration	[28,184,185]

BBB—blood–brain barrier; IL—interleukin; MCP-1—monocyte chemotactic protein 1; MMPs—matrix metalloproteinases; TNF-α—tumor necrosis factor-α.

#### 2.3.4. Neurotrophic Factors and Neuropeptides

Although the central nervous system has a limited ability to regenerate, neurogenesis is still possible in the subventricular zone and the subgranular zone of the dentate gyrus [205], although it declines with age. This process occurs in three steps [206]: (1) neural stem cell proliferation, (2) migration of neuroblasts and immature neurons, and (3) differentiation into mature neurons and synaptogenesis. Following ischemic stroke, neural stem cells migrate into the peri-infarct region and differentiate into neurons [207]. However, in an inflammatory environment lacking trophic factors, many of these immature neurons will not survive [208], which is why the role of various neurotrophic factors and the possibility of their upregulation has been investigated as a means of enhancing post-stroke recovery. Research has shown that insulin-like growth factor 1 (IGF-1), brain-derived neurotrophic factor (BDNF), and vascular endothelial growth factor (VEGF) are involved in neural stem cell proliferation, while stromal-derived factor 1, MCP-1, and MMPs 2, 3, and 9 mainly influence the migration of neuroblasts [209,210,211,212,213]. Angiogenesis, defined as the sprouting of preexisting vessels leading to the formation of new blood vessels, is closely intertwined with neurogenesis after stroke [214,215] and even precedes the neurogenic processes after an ischemic insult [216]. 

The link between neuroinflammation and neurogenesis is complex, with the former being able to both suppress and promote neurogenesis [206]. For example, short-term treatment of neural stem cells with IL-6 in vitro promotes cell division [217], while chronic astrocytic IL-6 expression impairs neurogenesis in the dentate gyrus of mice [218]. IL-17 released by astrocytes also promote neurogenesis via the NF-κB pathway [219]. Microglia have dual effects, being able to produce cytokines which impair neuronal survival as well as trophic factors which guide neural stem cell migration and support differentiation [220]. 

However, since most of these newly formed neurons die within 2-5 weeks [208], it is important to provide an environment rich in neurotrophic factors to promote their survival and integration into neuronal circuits. Unfortunately, most of these trophic factors are large molecules which hardly cross the BBB, making exogenous administration challenging. As such, several delivery strategies have been developed.

Central administration of IGF-1 prevented white matter lesions after hypoxic-ischemic insult in rat brain [221], but the molecule has limited bioavailability [222] and concerning metabolic and mitogenic effects [223]. A naturally cleaved tripeptide, glycine–proline–glutamate (GPE), retains the neuroprotective properties of the original molecule and can be safely administered intravenously 3-7 h after the ischemic insult [223], resulting in significantly improved outcome regardless of the animal’s age [224]. The peptide does not bind to IGF-1 receptors but activates NMDA receptors [225] and inhibits caspase-dependent and -independent apoptosis [224]. 

Extensive studies have reported on the neuroprotective effects of BDNF in various diseases of the CNS, from neurodegenerative ones to traumatic and vascular insults [226,227]. With important roles during development, BDNF exerts its neuroprotective functions after binding to tyrosine kinase receptors mainly through the phosphatidylinositol 3-kinase (PI3K)/Akt and the mitogen-activated protein kinase/extracellular-signal- regulated kinase (MAPK/ERK) pathways [228], which activate transcription factors such as AP1, NF-κB, and FOXOs, and induces genes encoding antioxidant enzymes, anti-apoptotic proteins, and proteins involved in ion homeostasis [229]. In addition, BDNF can induce local calcium influxes in neurites [230] and promote neurogenesis and dendritic outgrowth [138,231]. Increasing BDNF levels with medication, such as cilostazol [232], and delivering BDNF overexpressing fibroblasts or mesenchymal cells [233,234] or through exercise [235] improved post-stroke recovery. However, it also has a low transport rate across the BBB and, due to the involvement of tyrosine kinase B receptors in the regulation of metabolism and energy homeostasis [236], it carries the risk of weight loss. Improved delivery was achieved by incorporating polyethylene glycol (PEG) moieties [237], nanoparticle formulations [238], or fusion with viral-derived peptides [239]. 

The expression of nerve growth factor (NGF) and tyrosine kinase receptor A is rapidly upregulated following ischemia [240], promoting axonal sprouting and tissue repair [241]. However, in clinical trials, the systemic administration of NGF led to weight loss and myalgias [242].

Glial-cell-line-derived neurotrophic factor (GDNF) acts by binding to the extracellular glycosylphosphatidylinositol (GPI)-linked receptor, GFRα1, and the transmembrane tyrosine kinase, c-Ret [243], the expression of which is upregulated in the penumbral area [244]. Exogenous GDNF delivery yielded conflicting results: while short-term delivery proved neuroprotective [245], long-term elevated GDNF levels can exacerbate neuronal death [246]. Research has shown an enhanced neurogenesis mainly in the striatum [247], which is why this neurotrophin has been more extensively evaluated in Parkinson’s disease [248]. 

Other proteins and peptides have also been shown to promote tissue repair after stroke. One such protein is annexin A1, a calcium- and phospholipid-binding protein, with anti-inflammatory actions mediated through the G-protein-coupled formyl peptide receptor type 2 [249], which promotes microglial M2 polarization [250]. In a mouse stroke model, annexin A1 was found reduced at the onset of ischemia, followed by a return to normal levels after successful recanalization. Exogenous administration of the biologically active N-terminal peptide during reperfusion resulted in a diminished magnitude of tissue damage, likely due to a shift of microglia/macrophage cells toward M2 phenotypes via activation of the AMPK-mTOR pathway [251]. Leptin, a well-known hormone related to obesity, has also been shown to exhibit anti-inflammatory actions and to promote neuroprotection, cell differentiation, mitochondrial biogenesis, and angiogenesis [252], as well as to upregulate antioxidant defenses and anti-apoptotic proteins [253]. It exerts its effects through activation of the Janus kinase (JAK)-signal transducer and activator of transcription (STAT) [254], the Ras/extracellular signal-regulated kinase (ERK)1/2 [255], the adenosine monophosphate kinase (AMPK)-mammalian target of rapamycin (mTOR) [256], and the phosphoinositide-3 kinase (PI3K)/Akt pathways [257].

## 3. Therapeutic Approaches Focusing on Modulation of Neuroinflammation

Although promising in preclinical studies, the clinical trials targeting stroke immunology have not shown clear benefits despite strategies addressing both innate and adaptive immune responses [20]. The dual role of the inflammatory response, promoting cellular injury in the acute setting and restoration of the cellular functions during rehabilitation, is a major challenge for clinical trials targeting neuroinflammation. For example, microglial P2X4 receptors modulate the inflammatory response after ischemia, but in acute cerebral ischemia, the activation of these receptors leads to exacerbation of the inflammatory response, while their activation in chronic ischemia results in the release of BDNF, which supports synaptic plasticity. As such, microglial P2X4 receptor deletion is protective against acute cerebral ischemia but exacerbates behavioral abnormalities in the post-stroke rehabilitation process [258]. 

### 3.1. Therapies Targeting Microglia

Minocycline, a highly lipophilic antibiotic agent able to cross the BBB, was shown to be able to inhibit microglial activation, diminish the magnitude of neuronal apoptosis [259], decrease the rate of T cells migration, reduce the expression of chemokines and their receptors as well as the production of free radicals [260], and inhibit matrix metalloproteinases. In preclinical setting, minocycline diminished infarct size and hemorrhagic transformation [261] and did not interact with recombinant tissue plasminogen activator [262]. As such, minocycline administered within 24 h from stroke onset and continued for 5 days was evaluated in two phase 2 clinical trials alone or in combination with rtPA, which showed it to be safe, well tolerated, and potentially effective [263,264]. However, a third study, possibly underpowered, confirmed the safety but dismissed the efficacy of minocycline in acute ischemic stroke [265]. 

Among the receptors involved in microglial M1 polarization are TLR4 receptors [266]. In preclinical studies, the inhibition of TLR4 resulted in reduced infarct size and improved recovery in diabetic rats [267] A phase 1b/2a trial (NCT 04734548) is currently recruiting 151 patients eligible for endovascular treatment to evaluate the safety of inhibiting TLR4 receptors with ApTOLL [268]. 

In preclinical studies, the angiotensin II type 2 receptor agonist C21, administered 3 days after stroke onset, improved functional outcome by reducing neuroinflammation and shifting microglia toward the M2 phenotype [269,270]. 

Recent research focuses on influencing microglial polarization from M1 to M2 phenotype by using IL-4 or IL-33 in a preclinical setting [271,272]. 

### 3.2. Strategies for Blocking Neutrophil Infiltration

Several trials have focused on targeting leukocyte adhesion and transmigration as an anti-inflammatory treatment in acute ischemic stroke. A murine ICAM-1 antibody, enlimomab, interferes with leukocyte adhesion and was shown in experimental studies to reduce infarct size [273,274]. However, in a clinical trial, enlimomab administered within 6 h after stroke onset increased the rate of adverse events and significantly worsened the outcome of stroke patients [275]. 

A cellular adhesion molecule located on the leukocyte surface, L-selectin, has been targeted with a humanized monoclonal antibody (HuDREG200) alone or in combination with alteplase in a rabbit stroke model, but the results have not been disclosed [276]. 

Another humanized antibody targeting neutrophil adhesion, Hu23F2G, or LeukArrest, was tested in the HALT study, a phase 3 clinical trial which, although it was shown to improve the modified Rankin score at 3 months in a phase 2 study, was prematurely stopped by the sponsor due to lack of benefit suggested by an interim analysis [97]. 

Preclinical studies suggested that targeting CD11/CD18 for blocking neutrophil infiltration was beneficial only in transient ischemic stroke models and had no effect in permanent ones [277,278]. The ASTIN (Acute Stroke Therapy by Inhibition of Neutrophils) trial, tested a CD18 antagonist inhibiting polymorphonuclear adhesion and transmigration, namely UK-279,276, administered within 6 h from stroke onset on stroke outcome. Similar to the HALT trial, it was stopped early after futility criteria were reached [279].

Natalizumab, an anti-CD49b antibody, used in the immunomodulatory treatment of multiple sclerosis, has been tested in preclinical studies in ischemic stroke with conflicting results: one study showed no effect [280], while four studies suggested positive effects [281,282]. To further complicate the issue, a large preclinical trial concluded that the effectiveness of CD49d antibodies in stroke depends on infarct size and location, showing reduced leukocyte infiltration and lesion volume after permanent distal occlusion of the middle cerebral artery but not after transient occlusion of the proximal portion of the same artery [283]. In a clinical setting, natalizumab administered within 9 h after stroke onset (ACTION trial, NCT 01955707) did not reduce lesion size, but at 30 days, the outcome of patients in the treatment arm was better, an effect lost at the 90-day follow-up [284]. A second recently completed phase 2 clinical trial with 277 participants, ACTION II (NCT02730455), evaluated the effects of two different doses of natalizumab, given either within 9 h or between 9 and 24 h after stroke onset, on functional recovery [285].

### 3.3. Targeting Lymphocytes in Acute Ischemic Stroke

Since lymphocytes interfere in the delayed phase of ischemic injury, therapeutic strategies targeting these cells would theoretically have wide therapeutic windows. 

Preclinical studies suggested that blocking cerebral invasion of pro-inflammatory lymphocytes (TH1, TH17, γδT) would be neuroprotective [104,286]. Interferon β-1a, long used as immunomodulatory treatment in multiple sclerosis, prevents BBB disruption; reduces the proliferation and activation of T helper, γδ T cells, and CD8+ cells; and inhibits the production of pro-inflammatory cytokines [287]. The safety of four doses ranging from 11 to 88 mcg of interferon β-1a daily for 7 days in acute ischemic stroke was evaluated in a phase 1 clinical trial (NCT 00097318) in 60 patients, but no results have yet been published [268].

Regulatory T cells (Tregs) have been traditionally viewed as anti-inflammatory lymphocytes, but their therapeutic manipulation in different stroke models yielded divergent results. Treg depletion led to increased infarct sizes in three studies [288,289,290], and one study found a reduced infarct size in Treg-deficient mice [111], while other studies could not detect any influence on infarct size [111,291]. The different findings were explained by the various experimental models used and infarct sizes [20]. Apparently, Treg depletion is beneficial only in small infarcts in permanent occlusion models [113]. An opposite approach, transferring Tregs to wild-type animals or administering a CD28 superagonist, which expands the number of Tregs and amplifies their suppressive function, also resulted in divergent results, with the strategy either improving stroke outcome [292] or increasing infarct size [293]. One explanation of these divergent findings relates to the involvement of Tregs in microvascular thrombus formation and their contribution to secondary ischemia occurring after reperfusion, an event which does not occur in permanent focal ischemia stroke models [114]. 

Fingolimod, a drug approved for the treatment of multiple sclerosis, inhibits lymphocyte trafficking and infiltration of the central nervous system. Tested in models of transient and permanent focal ischemia, it did not influence lesion size and behavioral dysfunction [20]. A pilot phase 2 trial enrolling 22 participants (NCT02002390) who received 0.5 mg daily for 3 days suggested favorable effects on outcome, with smaller infarct sizes and reduced rates of bleeding [294], and demonstrated safety of the association of alteplase with fingolimod. Two other phase 2 studies are ongoing with fingolimod. One phase 2 trial aiming at enrolling 118 patients will assess outcome in patients receiving 0.5 mg fingolimod daily for 3 consecutive days started 1 h before thrombectomy (NCT 04675762), a trial in which standard stroke care also involves bridging between alteplase and mechanical thrombectomy, while a second phase 2 trial will assess collateral circulation in 30 patients randomized to receive either 0.5 mg fingolimod or placebo daily for 3 days starting 3 h prior to endovascular treatment (NCT 04629872) [268]. 

### 3.4. Other Drugs Which Modulate Neuroinflammation

1. Due to the potential of oxidative stress to elicit inflammatory responses, antioxidant molecules could act as anti-inflammatory agents as well. Although promising in vitro and in preclinical studies, most clinical trials failed or yielded inconclusive results [27]. A notable exception is edaravone, a free radical scavenger used since 2001 in Asian countries (mostly Japan) for the treatment of acute ischemic stroke with or without recanalization treatments, which was shown to improve neurological deficit without significant adverse events [295]. Given its potent antioxidant effect, the molecule was also approved for the treatment of amyotrophic lateral sclerosis in 2017 [42,296]. The drug may be combined with other antioxidant and anti-inflammatory molecules to increase its efficacy. A combination of 30 mg edaravone and 6 mg of d-borneol, known as Y-2, is able to scavenge hydroxyl radicals, nitric oxide, and peroxynitrite and inhibit the expression of TNF-α and IL-1. Its efficacy in acute ischemic stroke is evaluated in a phase 3 multicenter, randomized, double-blind, placebo-controlled trial (NCT 04950920), which is currently enrolling 900 participants [268].

2. Statins, aside from their lipid-lowering effects, also exhibit anti-inflammatory properties by altering the release of interleukins and TNF-α [297] and inhibiting platelet NOX2, thromboxane A2, and platelet isoprostane formation. Larger strokes have been shown to be associated with lower levels of TNF-α, IL-1β, P-selectin, and ICAM-1 as compared to lacunar strokes [298]. Lovastatin and atorvastatin showed promising results in animal models of acute ischemic stroke, and a phase 4 clinical trial, NCT02225834, which enrolled 50 patients who received 80 mg of Atorvastatin daily started within 48 h from stroke onset, demonstrated better clinical outcome in patients in the treatment arm [299]. In addition, chronic statin use before the ischemic event is associated with reduced stroke severity and improved functional outcome [300]. However, excessive and long-standing lowering of the low-density lipoprotein cholesterol fraction may be associated with cognitive decline [301]. 

3. Vinpocetine, a derivative of the alkaloid vincamine available in many countries as dietary supplement to enhance memory and cognition, reduces the release of pro-inflammatory cytokines and chemokines from microglia, endothelial cells, and vascular smooth muscle cells [302]. It can also antagonize atherosclerosis caused by a high-fat diet as well as injury-induced vascular remodeling [303]. Its effect in ischemic stroke was evaluated in a phase 2/3 clinical trial (NCT 02878772), which enrolled 60 patients who were randomized to receive 30 mg of vinpocetine daily for 14 consecutive days, starting within 48 h from stroke onset. The study is complete, but no results have yet been published [268].

4. RO27-3225, a melanocortin MC4 receptor agonist, reduces the expression of TNF-α, Bax, ERK, JNK, and caspase-3 and promotes improved functional recovery following ischemic stroke in gerbils [304]. 

5. Omega-3 polyunsaturated fatty acids prevent the release of cyclooxygenase 2, hypoxia-inducible factor 1α, and IL-1β, as well as the activation of NOS, thereby having therapeutic potential in stroke [305]. 

6. Pharmacological inhibition or genetic deletion of COX-2 limits the production of pro-inflammatory prostanoids and diminishes BBB damage in acute cerebral ischemia [306], but blocking COX-2 later after the ischemic event impairs the endogenous repair mechanisms [307]. In addition, COX-2 inhibitors are associated with an increased risk of ischemic vascular events and increased stroke mortality if used before stroke onset [308].

7. TNF-α receptor inhibition with R-7050 has recently been reported to decrease the activation of NF-κB and reduce levels of IL-6, thereby protecting against neurological deficits and edema in a rat model of permanent cerebral ischemia [309]. 

8. Inhibition of IL-1β receptor with recombinant human IL-1 receptor antagonist (IL-1Ra, or anakinra) was tested in the SCIL-STROKE trial based on the promising results of a previous trial in subarachnoid hemorrhage. Although the drug was safe and well-tolerated, it did not significantly improve the outcome of ischemic stroke patients [153].

9. MicroRNAs, members of the small, noncoding RNA superfamily, have been much studied in recent years. They are single-stranded RNA molecules comprising 18–25 nucleotides which inhibit the expression of protein-coding genes by degrading or inhibiting the translation of target mRNA [310,311]. MicroRNA-124 (miR-124) has been shown to protect neurons after ischemia by regulating the expression of key genes [312], to modulate neuroinflammation [313], and, after being transferred from neurons to astrocytes, to regulate the expression of glutamate transporter 1 [314]. Intracerebroventricular delivery of miR-124-loaded nanoparticles (NPs) stimulated neuronal differentiation of the subventricular stem cells after oxygen and glucose deprivation [315]. However, biological simulants of miR-124 introduced into ischemic neural progenitor cells inhibited their proliferation [316]. As such, the therapeutic or detrimental role of miR-124 in ischemic stroke is still under research. 

10. Transplantation of mesenchymal stem cells provides neuroprotection and immunomodulation and stimulates neurogenesis, astrogenesis, oligodendrogenesis, and angiogenesis, as well as the formation of new synapses [317]. They can be delivered intravenously [318], intra-arterially [319], or (in animal models) directly into the brain [320]. These cells then migrate into the damaged areas and differentiate into neurons and astroglial cells, also exhibiting paracrine activities, such as producing TGF-β and inhibiting the secretion of MCP-1 and leukocyte infiltration [321], as well as producing IL-2 and IL-6 [322]. Extracellular vesicles derived from mesenchymal stromal cells are safer to use, carrying a lower risk of vessel blockage and vascular thrombosis, having lower immunogenicity, and being more able to cross the BBB [317], which allow systemic transplantation. They contain proteins, lipids, nucleic acids, and membrane receptors. The miRNAs contained and subsequently transferred to cells in the brain parenchyma can easily be subject to genetic modifications to induce neurogenesis and angiogenesis, modulate the immune response, and inhibit apoptosis [323]. This approach has made it to clinical trials, with 20 clinical trials having been already carried out or that are ongoing [317]. Several technical issues have to be solved relating to the optimal route of transplantation, the adequate number of cells, determining the right timing of administration from stroke onset, and the frequency of delivery [317]. However, from the studies conducted so far, the strategy appears safe, without significant side effects, allowing administration after days to weeks following stroke onset and leading to functional improvement [324]. As for extracellular vesicles, although promising results were obtained in preclinical studies, no clinical trial has yet been conducted, but a phase 1/2 study with miR-124-enriched extracellular vesicles is listed as recruiting [268].

### 3.5. Reasons for Discrepancies

Many reasons for the discrepancies between preclinical and clinical trials can be discussed.

1. Most transient animal models of stroke use the mechanical artery occlusion, which causes thromboinflammation with subsequent microthrombosis, leading to brain injury. Clot-induced stroke models are less used, although they allow for determining the nature of the clot, whether platelet-rich or fibrin-rich [317]. Using these various models, it may well be that, for example, Tregs could target thromboinflammation and microthrombosis, thereby explaining the conflicting results of the studies performed. As such, immunomodulatory drugs should be evaluated in stroke models not associated with thromboinflammation.

2. In most animal models of transient arterial occlusion, complete occlusion is followed by complete reperfusion, a situation rarely achieved in human patients except for thrombectomized patients [20]. 

3. Many of the risk factors for stroke, such as smoking, age, alcohol consumption, stress, and age, cannot be adequately replicated in the laboratory, with most studies being performed on young animals. Moreover, although stroke predominates in aged women and inflammatory cells vary between genders, preclinical studies mainly use male animals [325]. 

4. One must not overlook the risk of infectious complications after stroke, which can significantly interfere with immunomodulatory drugs [326].

## 4. Concluding Remarks

Immunomodulatory therapy is a very appealing strategy for the treatment of acute ischemic stroke, both for patients treated with recanalization methods as well as for patients no longer eligible for reperfusion therapies. However, given the dual nature of neuroinflammation, further research is needed to establish the exact sequence and the stroke subtypes in which these strategies will yield positive results and lead to better functional outcomes. In our opinion, recanalization strategies will continue to be increasingly used for treating acute ischemic stroke, with extended therapeutic time windows through various methods (hypothermia, neuroprotectants) and a more careful selection of eligible patients (trying to identify the presence of penumbra through MRI techniques). Recanalization would be administered concomitantly with a cocktail of antioxidant molecules to diminish the magnitude of oxidative stress induced by reperfusion. In the subacute phase, neuroinflammatory pathways could be modulated to enhance the proliferation and migration of stem cells and improve recovery. From the studies performed so far, it appears that these strategies have an extended time window. It seems more plausible that using endogenous repair mechanisms, such as stem cell transplantation or genetically manipulated extracellular vesicle delivery, would have a better outcome than simply delivering exogenous molecules. To avoid rejection, autologous bone marrow cells could be harvested from the patient in the acute phase.

## Figures and Tables

**Table 1:** Pathophysiology of cerebral ischemia and reperfusion injuries.

Time Course	Pathophysiological Mechanisms
Acute phase (minutes–hours)	Reduced cerebral blood flow with diminished oxygen and glucose delivery Anaerobic metabolism and lactic acidosis Failure of ATPase, cellular depolarization, increased intracellular ion influx Release of neuromediators (excitotoxicity) Increased expression of stress signaling genes
Subacute phase (hours–days)	Increased production of ROS Apoptosis Expression of adhesion molecules Microglial activation and leukocyte infiltration of the brain Release of pro-inflammatory mediators Increased activity of proteolytic enzymes and damage of BBB and endothelium
Chronic phase (days–weeks)	Release of trophic factors (BDNF, IGF, GDNF) Neurogenesis, angiogenesis, synaptogenesis Activation of stem cells

ATPase—adenosine triphosphatase; ROS—reactive oxygen species; BBB—blood–brain barrier; BDNF—brain-derived neurotrophic factor; IGF—insulin-like growth factor; GDNF—glial-derived neurotrophic factor.
